# Multi-view gene panel characterization for spatially resolved omics

**DOI:** 10.1093/bib/bbaf478

**Published:** 2025-10-04

**Authors:** Daniel Kim, Wenze Ding, Akira Nguyen Shaw, Marni Torkel, Cameron J Turtle, Pengyi Yang, Jean Yang

**Affiliations:** Sydney Precision Data Science Centre, The University of Sydney, Sydney, NSW, Australia; Computational Systems Biology Unit, Children’s Medical Research Institute, Westmead, NSW, Australia; Charles Perkins Centre, The University of Sydney, Sydney, NSW, Australia; School of Medical Sciences, Faculty of Medicine and Health, The University of Sydney, Sydney, NSW, Australia; Sydney Precision Data Science Centre, The University of Sydney, Sydney, NSW, Australia; School of Mathematics and Statistics, The University of Sydney, Sydney, NSW, Australia; Sydney Precision Data Science Centre, The University of Sydney, Sydney, NSW, Australia; School of Medical Sciences, Faculty of Medicine and Health, The University of Sydney, Sydney, NSW, Australia; Sydney Precision Data Science Centre, The University of Sydney, Sydney, NSW, Australia; School of Medical Sciences, Faculty of Medicine and Health, The University of Sydney, Sydney, NSW, Australia; Sydney Medical School, Faculty of Medicine and Health, The University of Sydney, Sydney, NSW, Australia; Royal North Shore Hospital, St. Leonards, NSW, Australia; Fred Hutchinson Cancer Center Seattle, Seattle, WA, United States; Sydney Precision Data Science Centre, The University of Sydney, Sydney, NSW, Australia; Computational Systems Biology Unit, Children’s Medical Research Institute, Westmead, NSW, Australia; Charles Perkins Centre, The University of Sydney, Sydney, NSW, Australia; School of Medical Sciences, Faculty of Medicine and Health, The University of Sydney, Sydney, NSW, Australia; School of Mathematics and Statistics, The University of Sydney, Sydney, NSW, Australia; Sydney Precision Data Science Centre, The University of Sydney, Sydney, NSW, Australia; Charles Perkins Centre, The University of Sydney, Sydney, NSW, Australia; School of Mathematics and Statistics, The University of Sydney, Sydney, NSW, Australia

**Keywords:** gene panel, spatial transcriptomics, large language models, multi-objective optimization

## Abstract

Spatially resolved transcriptomics has revolutionized the study of complex tissues by enabling cellular and subcellular resolution. However, targeted spatial technologies depend on pre-selected gene panels, which are typically curated based on prior biological knowledge or specific research hypotheses. While existing methods often focus on optimizing for cell type identification, we argue that effective panel design should also account for transcriptional variation, pathway-level coverage, and minimal gene redundancy. To meet these broader criteria, we developed a two-part framework: (i) *panelScope*, a gene panel characterization platform that characterizes panels from multiple perspectives, allowing for holistic comparisons of gene panels for custom panel design; and (ii) *panelScope-OA*, a genetic algorithm that integrates these characterization metrics into a multi-loss function to automate panel optimization. We applied *panelScope* and *panelScope-OA* to characterize nine panels across four datasets. Notably, computationally constructed gene panels performed competitively in capturing major cell types when compared to our in-house manually curated panel. However, refined manual curation offered distinct advantages, particularly in capturing minor cell types. Our results demonstrate the utility of *panelScope* and *panelScope-OA* by offering quantitative and multi-dimensional insights to support the design of panels tailored to diverse research needs.

## Introduction

The emergence of spatially resolved transcriptomics (SRT) has transformed our ability to quantify gene expression *in situ* while preserving the native tissue environment. This technological leap has revealed which genes are expressed and where they are expressed within complex tissues, which is critical for elucidating cell–cell interactions, tissue heterogeneity, and the architectural nuances that govern disease progression and therapeutic response. Consequently, SRT has enabled insights that would be difficult or impossible to achieve using conventional sequencing alone [1, 2].

There are a variety of SRT techniques, including fluorescence *in situ* hybridization (FISH)-based methods, such as seqFISH+ and MERFISH, which directly label and visualize transcripts within tissue sections [3, 4]. In contrast, spot-level ribonucleic acid (RNA)-sequencing techniques, such as SLIDE-seq and high-definition spatial transcriptomics (HDST), utilize barcoded bead arrays to capture RNA while retaining spatial coordinates [5]. These SRT platforms differ significantly in their capabilities: sequencing-based approaches (e.g. SLIDE-seq, HDST) can capture whole-transcriptome data (un-targeted transcriptomics), while *in situ* hybridization methods generally focus on a targeted subset of genes of interest (targeted transcriptomics) [6]. Targeted spatial transcriptomics is often employed when researchers wish to focus on a specific set of genes and do not require whole-transcriptome data. By sequencing a smaller subset of genes, they can achieve higher sensitivity and accuracy, which is particularly valuable for detecting lowly expressed genes. However, targeted spatial transcriptomics requires the careful design of a gene panel tailored to specific research objectives.

Several computational methods have been developed to facilitate panel design and can be broadly categorized into two groups. The first category, which is imputation-driven approaches, selects highly variable genes and ranks them by the extent to which they recapitulate transcriptional variability [7–11]. Examples in this category include *geneBasis*, which selects genes that best preserves the transcriptional manifold of the reference data [9] and *PERSIST*, a deep-learning algorithm, which selects genes that best predicts the full single-cell expression profile of a reference dataset [11]. The second category, classification-driven methods, concentrates on genes whose expression patterns best discriminate cell types or other discrete labels [12–16]. Examples in this category include *gpsFISH*, which couples a genetic algorithm with cross-validation to choose genes that maximize cell-type-classification accuracy [17], *scGIST*, a deep-learning-based approach [16] and *panelScope/panelScope-OA*, our current work. Most existing algorithms optimize panel accuracy alone, giving little attention to other desirable properties such as minimizing redundancy or ensuring pathway coverage. A notable exception is *Spapros*, which incorporates additional criteria, beyond cell type classification accuracy, into its panel design process [10].

What remains lacking is a systematic approach to characterize whether a chosen panel meets the practical needs of a study. The characteristics that make a ‘good’ panel, for example minimal redundancy or broad pathway representation, vary with scientific aims. A unified, quantitative framework for gene-panel characterization and design would allow researchers to align design choices with their specific objectives and to compare alternative panels for custom panel design. To this end, we present *panelScope*, a framework that uses diverse metrics to characterize gene panels, helping researchers assess their suitability for specific study objectives. *panelScope* generates multi-view summaries of each panel, evaluating aspects such as cell type coverage, biological pathway enrichment, and feature redundancy. These metrics also serve as loss functions in a customizable genetic algorithm for panel design, *panelScope-OA*, allowing users to weight categories based on their goals.

## Materials and methods

### Datasets

#### Dataset 1—AML dataset

We illustrate our *panelScope* framework using a single cell RNA-seq dataset of bone marrow aspirates from 19 Acute Myeloid Leukaemia (AML) patients and a healthy individual [18]. Both healthy hematopoietic differentiation and diseased myeloid states are represented in this dataset. We preprocessed the data by removing any genes with less than a total of 10 counts, cells with less than a total of 200 counts, and cell types with less than 10 cells. The final dataset included 39 146 genes, 3437 cells, and 37 cell types, as annotated by the original authors.

#### Dataset 2—lymph node dataset

To demonstrate the utility of our spatial metrics we used the 5 k Xenium Panel of human lymph nodes by 10 Genomics, available at: https://www.10xgenomics.com/resources/datasets (Accessed 10 October 2024). This dataset includes 4624 genes, 708 983 cells, and 47 977 580 regions. Given the dataset’s size, we downsampled the original data to include 70 487 cells while retaining all 4624 genes without applying any additional filtering. This was to ensure computational efficiency when calculating the spatial features.

### Gene panel curation

We curated eight gene panels to apply our framework and illustrate how different panel properties can be characterized. Our first example uses the AML dataset and features eight gene panels generated through various strategies, including expert curation, random selection, computational methods, and the use of large language models.

#### Random

We selected a random set of genes (*n* = 160) from the dataset.

#### Stably expressed genes

A set of stably expressed genes (SEGs), previously identified [19], (*n* = 160). We selected the top n genes according to the stability scores provided in the original study.

#### Expert-curated

An in-house manually-designed AML gene panel (*n* = 160). Potential markers for each cluster were identified via non-negative matrix factorization. Markers were selected based on the specificity for each cluster, as verified by visual inspection of gene expression across dimension reduction plots.

#### Immune Oncology

This is the Immune Oncology gene panel designed by 10X Genomics available from https://www.10xgenomics.com/products/xenium-panels (*n* = 380).

#### geneBasis

Panel selection using *geneBasis* after its initial gene screening function ‘retain_informative_genes’, all parameters were set to default values (*n* = 117).

#### gpsFISH

We conducted panel selection by following *gpsFISH’s* vignette (*n* = 117).

#### Spapros

Panel was selected following *Spapro*s’s vignette (*n* = 200).

#### panelScope-OA

Optimization algorithm based on our developed metrics (see Supplementary Material 1 for details) (*n* = 200).

#### o1

To explore the capabilities of *o1*, we crafted two distinct prompts for designing two separate AML-specific gene panels (Supplementary Material 1). Each prompt instructed *o1* to maximize the characteristics set forth in our framework. Prompt 1 specified the cell types expected in AML (*o1*), while prompt 2 listed all 42 cell types present in the AML dataset (*o1+*). Both panels contained 200 genes each.

Our second example uses the lymph node dataset described above to demonstrate the spatial metrics of *panelScope*. We excluded the expert-curated panel from this experiment as it requires extensive manual effort and time. The two prompts used for this dataset can be found in the Supplementary Material (Supplementary Materials 2 and 3).

### *panelScope* characterization metrics

#### Feature specificity

This category evaluates how well a gene panel distinguishes groups of interest (e.g. cell types or states). We train a random forest classifier using the genes in a given panel; informative panels yield higher classification performance, measured by balanced accuracy and group-stratified accuracy. We also define additional metrics for this category, which include (i) gene specificity score ($gsc$), which aims to quantify the specificity of an individual gene for a given cell type (Supplementary Material 1), (ii) panel entropy score, to quantify the overall gene panel specificity for the cell types in a dataset by measuring spatial autocorrelation of the $gsc$ scores after hierarchical clustering, weighted by the proportion of zeros (Supplementary Material 1), and (iii) variation recovery score, to measure a gene panel’s ability to recover transcriptional variation (Supplementary Material 1).

#### Feature diversity

The key metric for this category is the Feature diversity score, which aims to capture the informativeness of a gene panel by assessing panel redundancy by computing pairwise Spearman’s correlations among all genes for a given panel (Supplementary Material 1).

#### Biological inference

To capture pathway coverage and diversity, we report the number and proportion of panel genes enriched in significant pathways, total pathway count, maximum q-value (false discovery rate), and a pathway diversity score reflecting the diversity of pathways captured (Supplementary Material 1). We also assess cell–cell interaction potential by classifying genes as ligands or receptors using CellChat annotations [20], and sum the counts in each category to evaluate enrichment (Supplementary Material 1).

#### Spatial information

We quantify the amount of spatial information within a gene panel by calculating Moran’s I for each gene in a panel to quantify spatially variable genes (Supplementary Material 1). We also calculate the correlation of the gene expression values for a panel’s genes between cells and their nearest neighbors to quantify nearest neighbor correlation (Supplementary Material 1).

#### Forward-compatibility

A crucial aspect of gene panel design is ensuring the selected genes remain relevant across various conditions, including treatments, experimental groups, or perturbations, termed ‘future versatility’ or ‘forward compatibility’. To evaluate this, we employed the GEARS perturbation simulator [21], trained on multiple public single-cell perturbation datasets [22–25]. Datasets were processed according to GEARS guidelines, involving normalization and quality filtering. We then used this model to simulate perturbations of each gene in the training set, focusing on overlapping genes between the training data and our panel. We summarized each gene’s perturbation impact by calculating a perturbation score, reflecting the number and magnitude of influenced genes (Supplementary Material 1).

#### Gene importance score and overall score

To provide a comprehensive characterization of the gene panels, we compute an importance score for each gene within a panel, which reflects the gene’s contribution to a panel across the defined categories. We also calculate a weighted overall score which evaluates the performance of a gene panel across the categories to allow for a holistic comparison between a set of gene panels (Supplementary Material 1).

### panelScope-OA

Here, we framed gene panel design as a hyperparameter optimization task, where each panel’s position is a hyperparameter and all genes are candidates. Starting from a random panel, the algorithm iteratively replaces genes to improve performance based on an objective function: the normalized, equally weighted sum of metrics defined above (scored 0–1).

To explore the panel space, we evaluate multiple candidate panels per iteration, generating and evaluating panels from an initial set of genes. Importantly, *PanelScope’s* modular design allows easy substitution of components (Supplementary Material 1).

## Results

### Multi-view gene panel characterization presents a comprehensive view for a pre-defined gene panel

**Figure 1:**
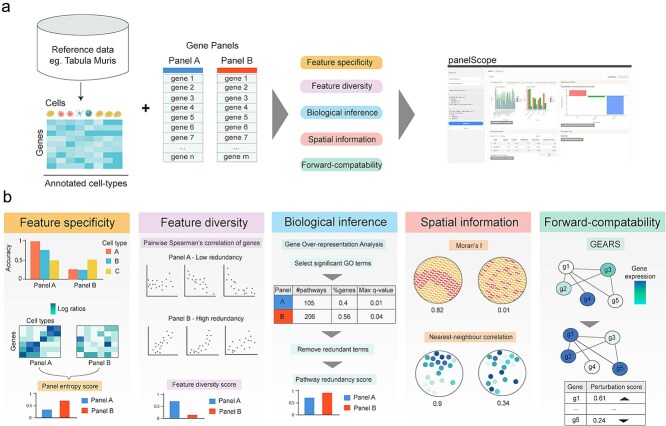
a) Workflow of gene panel characterization using *panelScope*. (b) Description of the five categories of metrics used to characterize a given gene panel: feature specificity, feature diversity, biological inference, spatial information, and forward-compatibility, which we demonstrate are robust to panel size (Supplementary Fig. 1).

We present *panelScope*, a modular framework for evaluating gene panels across five metric categories, accessible through a user-friendly web interface (Fig. 1a and b). It provides detailed gene- and panel-level scores, enabling users to identify informative genes, compare panels, and refine designs based on specific strengths and weaknesses. To complement this, we developed a genetic algorithm *panelScope-OA* that designs and optimizes panels using *panelScope*’s metrics. Users can customize the optimization algorithm by assigning weights to four core metric categories, allowing panel evaluation to align with diverse research priorities.

### Application of *panelScope* to AML to illustrate cell type-specificity of panels

**Figure 2:**
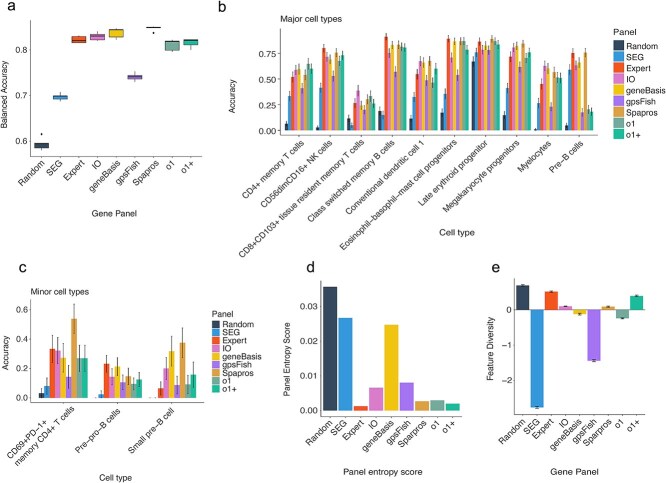
(a) Boxplots of balanced accuracy, measuring each panel’s overall ability to capture both major and minor cell types. (b) Barplot of accuracy for major cell types. Error bars show ±1 standard error. (c) Same as panel B, but for minor cell types. (d) Panel entropy redundancy score; lower values indicate greater gene specificity and are more desirable. (e) Feature diversity scores for each panel; positive values reflect greater diversity, while negative values indicate redundancy.

As a case study we applied our framework to design a panel guided by a dataset specific to acute myeloid leukemia [18]. We assessed the performance of each panel across the categories, starting with their ability to classify cell types in the reference dataset using genes from the respective panels. Classification performance was quantified using balanced accuracy and accuracy (Figs 2a–c).

The *expert-curated*, *geneBasis*, *gpsFish*, *IO*, *Spapros*, and *o1* panels achieved similarly high balanced accuracy, indicating strong overall performance. In contrast, *Random* and *SEG* panels performed worse due to lower specificity, though both still scored above 0.5—suggesting the presence of some cell type–specific genes, even without deliberate selection.

This was especially notable for the *SEG* panel, composed of SEGs typically linked to uniform expression and core cellular functions like glycolysis [19]. However, certain SEGs such as BIRC5 are differentially expressed in cancer [24, 25], supporting the idea that context-dependent perturbations can confer unexpected specificity—likely contributing to the *SEG* panel’s moderate performance.

To further dissect cell type specificity, we evaluated each panel’s ability to capture both major and minor populations (Figs 2b and c). While all panels reliably identified major cell types (e.g. aberrant erythroid cells; accuracy >0.8), *Random* and *SEG* panels underperformed for rare populations—highlighting the importance of targeted panel design for low-abundance cell types.

We also analysed feature diversity (Fig. 2e), which quantifies the proportion of non-redundant (orthogonal) gene pairs in each panel based on Spearman’s correlation. Higher scores indicate less redundancy. The *SEG* panel showed the greatest redundancy, consistent with strong co-expression among housekeeping genes. In contrast, the *Random*, *Expert-curated*, and *o1+* panels exhibited higher feature diversity. While the *Random* panel’s high score may seem counterintuitive, random gene selection often results in weakly correlated gene pairs, inflating its diversity metric.

### Feature specificity: panels with orthogonal information (e.g. *random* and *expert-curated* panels) exhibited higher diversity compared to *SEG*, which contained highly correlated genes

**Figure 3:**
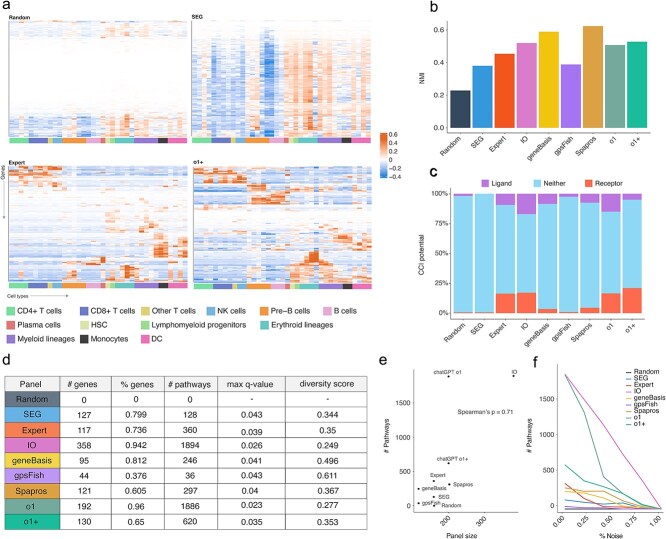
(a) Heatmaps of gscs (genes as rows, cell types as columns). Top: low-performing panels (*Random*, *SEG*); bottom: high-performing panels (*Expert*, *ChatGPT o+*). (b) NMI scores quantifying how much transcriptional variation each panel retains. (c) Stacked barplot showing counts of ligands, receptors, and other genes per panel. (d) Table summarizing pathway metrics per panel (adjusted *P* < .05): # genes: number of genes from panel that were enriched in significant pathways; % genes: proportion of genes in panel that were enriched in significant pathways; # pathways: number of significant pathways for a given gene panel; max q-value: maximum q-value (false positive rate) out of all significant pathways; diversity score: One minus the relative change in number of significant pathways when removing pathways with a Jaccard similarity index >0.7. (e) Scatter plot of number and proportion of enriched genes versus pathway count. (f) Scatter plot showing how pathway count changes as random (noise) genes are added to each panel.

To assess feature specificity, we developed a panel entropy score reflecting the proportion of non-cell type-specific genes (Fig. 2d). This involved computing a $gsc$ based on the log ratio of gene expression proportions between target and other cell types. Positive $gsc$ values indicate higher specificity. Hierarchical clustering of $gsc$ scores revealed patterns of gene specificity, with distinct clusters indicating low entropy (high specificity) and dispersed patterns indicating high entropy (low specificity) (Fig. 3a). These patterns were quantified using a weighted Moran’s I, adjusted by the proportion of low-specificity genes. As expected, *Random* and *SEG* panels had low specificity (high entropy). We also measured transcriptional variation using normalized mutual information (NMI), finding that the *Spapros* and *geneBasis* panel best captured global transcriptional diversity (Fig. 3b). Although NMI is theoretically independent of panel size, its estimate can fluctuate markedly when calculated from small gene sets due to sampling variability. Consequently, NMI values for very small panels should be interpreted with caution.

### Biological inference

The Biological Inference category evaluates the biological relevance of each panel by assessing ligand and receptor content (Fig. 3c) and pathway enrichment (Fig. 3d). As expected, *Random* and *SEG* panels had the fewest ligands and receptors. In contrast, the *Expert-curated*, *o1*, and *o1+* panels had the highest proportions (26%, 31%, and 26%, respectively), outperforming algorithm-based methods like *geneBasis* (11%) and *gpsFISH* (0.3%). This advantage may arise from the reasoning capabilities of large language models and their exposure to vast amounts of pre-training data, which seem to enhance ligand and receptor selection.

We assessed pathway information in gene panels using metrics such as the number and proportion of enriched genes, total significant pathways, maximum q-value, and pathway diversity score (Fig. 2d). Higher diversity scores indicate broader, non-overlapping pathway coverage. Panel size correlated with the number of significant pathways (Spearman’s ρ = 0.71) (Fig. 3e), but this depended on biological relevance—random panels showed no enrichment. Adding random genes reduced pathway enrichment across all panels, confirming that biological coherence, not just size, drives enrichment (Fig. 3f).

### Forward compatibility and spatial information

We include two optional evaluation categories—‘Forward Compatibility’ and ‘Spatial Information’—which can be applied when relevant data (e.g. spatial transcriptomics) are available. These categories are designed to extend panel utility beyond basic transcriptomic profiling.

#### Forward compatibility

**Figure 4:**
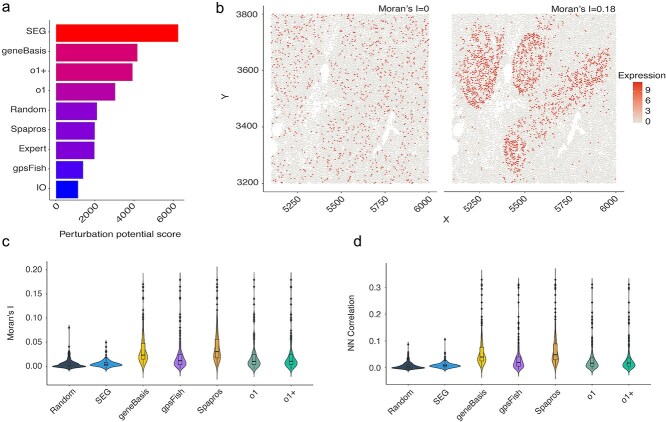
(a) Panels ranked by perturbation potential score—higher scores indicate a greater likelihood of genes being perturbed. (b) Spatial expression of two genes with contrasting spatial autocorrelation: PROCR (random, low Moran’s I) versus CXCL13 (clustered, higher Moran’s I). Red = high expression; white/gray = low/no expression. (c) Violin plots of Moran’s I across all panels, showing distribution and spread of spatial autocorrelation per gene. (d) Violin plots of nearest-neighbor (NN) correlation as an alternative measure of spatial clustering.

This category identifies panels likely to have broad regulatory influence when perturbed. Using GEARS, a simulation-based tool, we estimated perturbation potential across five datasets with diverse conditions (Fig. 4a). Higher scores indicate genes whose perturbation affects many downstream targets. Notably, the *SEG* panel scored highest—likely due to the central role of SEGs in essential cellular functions [26], where perturbation can trigger widespread effects including cell death and transcriptional reprogramming [27].

#### Spatial information

To assess spatial relevance, we evaluated spatial autocorrelation (Moran’s I) and Nearest Neighbor Correlation (Fig 4b–d). Panels with high spatial scores contain genes with localized expression patterns, which are valuable for spatial transcriptomics. *Random* and *SEG* panels showed lower spatial enrichment, reflecting their lack of spatial optimization. In contrast, *geneBasis* and *Spapros* scored higher, likely due to their selection for transcriptional variation, which overlaps with spatially distinct cell types [28, 29]. Because these metrics are gene-specific, they also support manual refinement of panels for spatial studies.

### *panelScope* leverages the multi-view panel characteristics to create gene panel *panelScope*

**Figure 5:**
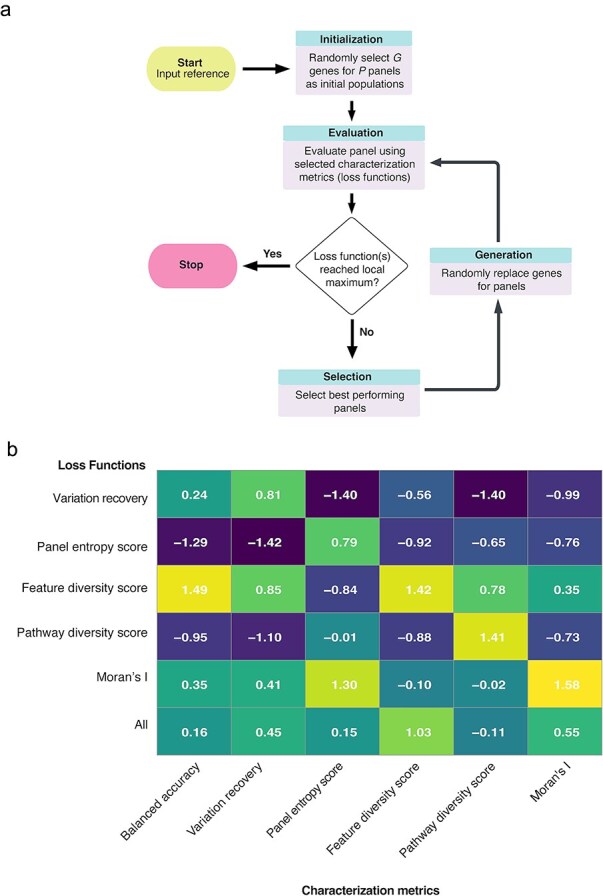
A) Schematic of the gene panel selection algorithm. Starting from 50 randomly chosen panels, an iterative optimization (default: 5000 steps) evaluates and replaces underperforming panels using scores for feature diversity, pathway diversity, panel entropy, spatial specificity, and variation recovery. (b) Heatmap of standardized scores for six optimized panels, each selected using a different loss function. Rows represent loss functions; columns represent evaluation metrics (e.g. balanced accuracy, pathway diversity). Color saturation reflects performance; panel entropy scores were negated for clarity.

The gene panel selection method *panelScope*-OA is summarized in Fig. 5a. The algorithm employs an iterative optimization process beginning with an initial random selection of 50 gene panels, each containing 200 genes. Each iteration (default: 5000 iterations) involves evaluating panels using predefined metrics, selecting top performers, and generating new panels by replacing lower-performing ones. This process continues until convergence criteria or maximum iterations are reached, after which the highest-scoring panel is chosen. This genetic algorithm approach effectively balances exploration and refinement, consistently improving panel performance through successive iterations.

We categorized characterization metrics into five groups: feature specificity, feature diversity, biological inference, spatial information, and forward compatibility, monitoring optimization progress individually and collectively (Supplementary Figs 2 and 3). Comparing optimization strategies revealed that variation recovery and panel entropy scores provided category-specific information, whereas optimization via feature diversity consistently yielded broader improvements. Consequently, the feature diversity-focused panel delivered enhanced performance across multiple metric categories (Fig. 5b). In addition, we compared *panelScope-OA* against several methods tested here (Supplementary Figs 4 and 5).

## Discussion

Here, we have presented a comprehensive gene panel characterization framework and algorithm spanning cell type specificity, feature diversity, biological inference, spatial information, and forward compatibility. It includes two complementary tools: *panelScope*, an interactive web tool that quantifies panel characteristics for more informative design decisions; *panelScope*-*OA*, an optimization algorithm leveraging differential weighting of panel features for customizable panel design. Currently, no quantitative framework exists to assess gene panel informativeness, making design decisions challenging. *panelScope* addresses this by evaluating panels across five categories, providing multiple views of panel characteristics, and allowing for researchers to refine panels accordingly, ensuring they meet specific objectives.

While the effort in the community to date has focused on determining the optimal gene panel, it is important to recognize that the optimality of a gene panel is criteria-dependent. The optimality of a gene panel depends on the specific research objectives it is designed to address. For instance, a panel could select genes that are key markers for immune cell types in a particular cancer, such as CD8+ T cells or tumor-associated macrophages (TAMs) in lung cancer, to assess immune infiltration and cancer immunotherapy targets [30]. Alternatively, a panel could focus on signaling pathways like PI3K-AKT or RAS–RAF–MEK, which are relevant across multiple cancers, where capturing diverse pathways is desirable [28, 29]. Naturally, given the limited number of genes that can be selected, there is a trade-off between these objectives. Thus, our interactive web tool enables users to explore these trade-offs, relating this information to the prioritization of specific objectives over others, allowing them to make quantitatively informed decisions on panel design. This flexibility ensures that researchers can tailor their gene panels to meet their specific research goals, whether they are focused on addressing multiple research questions or homing in on specific areas of interest.

An important factor in designing a gene panel is ensuring that the selected genes are likely to be forward compatible, meaning they will be differentially expressed across various treatments, experimental groups, or perturbation conditions. This aspect of ‘future compatibility’ ensures the panel remains relevant over time, allowing it to adapt to evolving research needs and experimental designs. Our current strategy leverages simulations with the GEARS framework, though this component can be replaced with improved simulation strategies in the future. Emerging methods for perturbation prediction utilize existing datasets from drug and genetic perturbations to forecast cellular responses to unseen changes. These approaches incorporate recent advances in AI and multi-omics data, such as scGen, which uses auto encoders for latent space shifts; CellOT, which applies optimal transport theory; CellOracle, which integrates chromatin accessibility for genetic perturbations; and PerturbNet, which uses both genetic and chemical structure data [31–34]. As these algorithms improve in the coming years, the metrics associated with this category in *panelScope* will also evolve, providing increasingly accurate and refined predictions for future compatibility.

Although *panelScope* was designed to characterize and evaluate gene panels designed for targeted spatial transcriptomics, many of the characterization categories are not dependent on spatial statistics, thus making *panelScope* generalizable to any technology requiring targeted gene panel selection, such as nCounter or Ion AmpliSeq. This may seem counterintuitive, however spatial metrics have limited applicability for characterizing gene panels due to the scarcity of comprehensive spatial datasets that can be leveraged for this purpose. Instead, we have intentionally designed multiple spatially agnostic metrics that can achieve comparable goals, while taking advantage of the vast availability of single-cell transcriptomic data. However, overtime, this will change as more spatial datasets become available. Importantly, *panelScope* can be extended to incorporate multi-omics data, such as proteomics, as these other molecular platforms often offer complementary information that will enhance the comprehensiveness of the panel selection. This would allow the framework to capture a broader and more comprehensive view of biological characteristics.

The genetic algorithm is a popular method utilized for feature selection [35]. Going forward, the accompanying genetic algorithm for gene panel design can be further improved through the incorporation of reinforcement learning techniques. Although much more computational resources would be needed, reinforcement learning would enable the framework to learn from feedback throughout the selection process, continuously refining gene panels based on the selected evaluation metrics. This approach would provide a more adaptive and personalized optimization process, where the system would evolve and improve with each iteration based on real-time feedback.

## Conclusion

In conclusion, we have developed a comprehensive framework for gene panel characterization across multiple views, *panelScope*. This is crucial for allowing transparent comparisons between gene panels and enables researchers to make informed decisions when designing gene panels. In addition, we have used these metrics to develop a genetic algorithm for panel selection *panelScope-OA*. Unlike other panel selection methods, *panelScope-OA* efficiently balances exploration and exploitation, continually refining gene panel selection while avoiding premature convergence.

**Key Points**

- *panelScope* introduces novel metrics across multiple categories for comprehensive gene panel characterization.
- *panelScope* provides a flexible characterization framework and *panelScope-OA* supports both manual and automated approaches for custom panel design.
- The optimization algorithm, *panelScope-OA*, goes beyond capturing cell types of interest, enabling a more holistic, context-aware approach to gene-panel development.

## Supplementary Material

Supplementary_figure_1_bbaf478

Supplementary_figure_2_bbaf478

Supplementary_figure_3_bbaf478

Supplementary_figure_4_bbaf478

Supplementary_figure_5_bbaf478

Supplementary_material_1_bbaf478

Supplementary_material_2_bbaf478

Supplementary_material_3_bbaf478

## Data Availability

The *panelScope* web application can be accessed at http://shiny.maths.usyd.edu.au/panelScope/. *panelScope-OA* can be accessed at https://github.com/SydneyBioX/panelScope/tree/main.
