# Supplementary material for: Multi-view gene panel characterization for spatially resolved omics
Source: Brief Bioinform. 2025 Oct 4;26(5):bbaf478. doi: 10.1093/bib/bbaf478 (PMC12495993; doi:10.1093/bib/bbaf478)
Supplement: Supplementary_figure_1_bbaf478 [file supplementary_figure_1_bbaf478.pdf]

Supplementary Figure 1

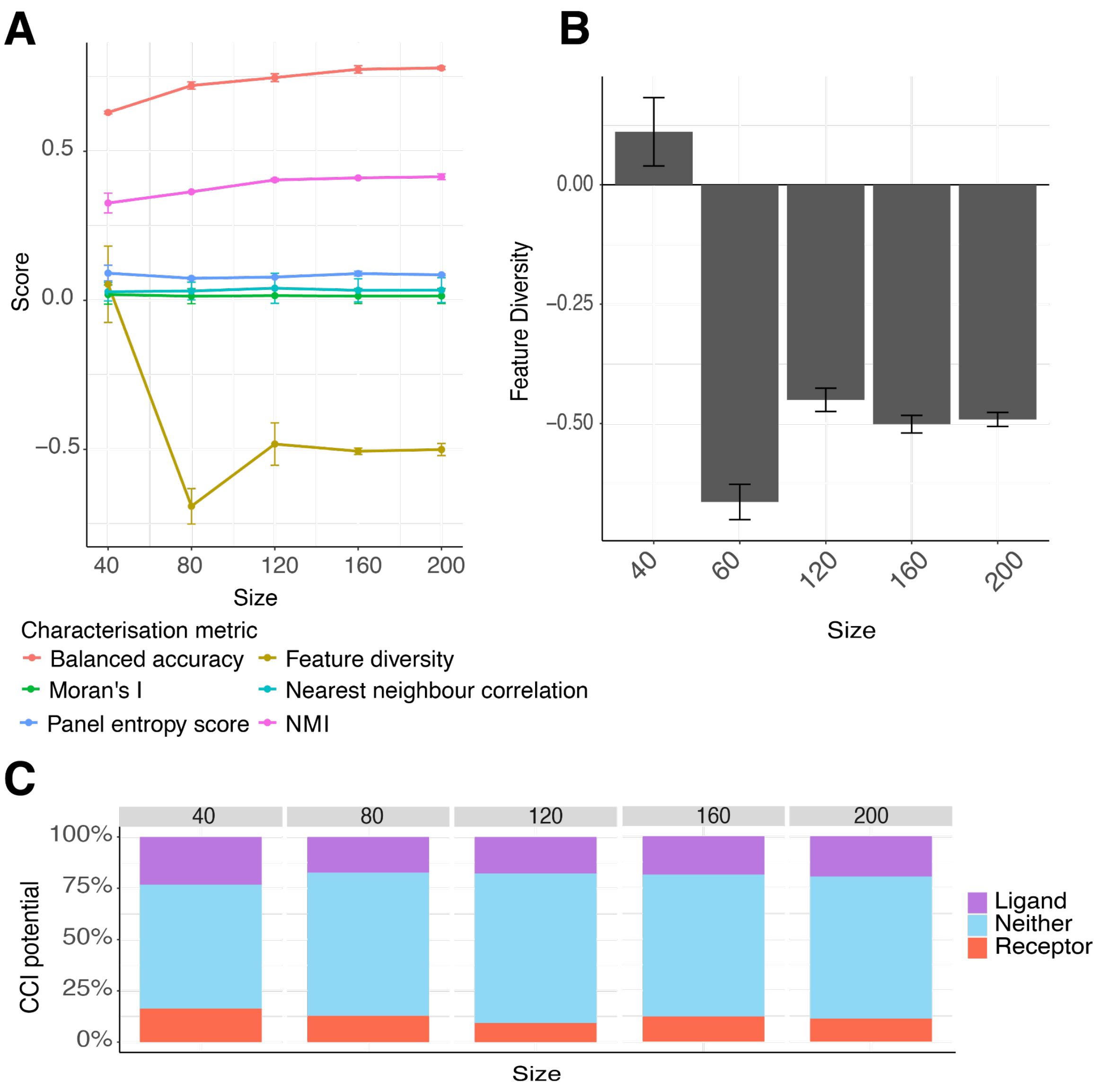

Supplementary Figure 1. **A) Panel-size sensitivity test.** Starting from a 200-gene *o3* panel, we repeatedly sampled fractions of the panel and recalculated all *panelScope* metrics. We did this five times to capture variability. Error bars represent +/- one standard deviation from the mean. Most metrics remained stable across panel sizes, except for Feature diversity, which showed some sensitively to panel size **B) Woolf-adjusted uncertainty for Feature diversity.** To quantify size dependence, we accompany the Feature diversity score with Woolf's standard error of the underlying log-ratio; the error bars shown quantify the sampling variance expected for a subset of a given size and represent one standard error. **C) Ligand/receptor content.** Applying the same subsampling experiment to the proportion of ligand and receptor genes revealed no systematic dependence on panel size, confirming that this metric is size-robust. This analysis was performed once.
