# Supplementary material for: Multi-view gene panel characterization for spatially resolved omics
Source: Brief Bioinform. 2025 Oct 4;26(5):bbaf478. doi: 10.1093/bib/bbaf478 (PMC12495993; doi:10.1093/bib/bbaf478)
Supplement: Supplementary_figure_2_bbaf478 [file supplementary_figure_2_bbaf478.pdf]

Supplementary Figure 2

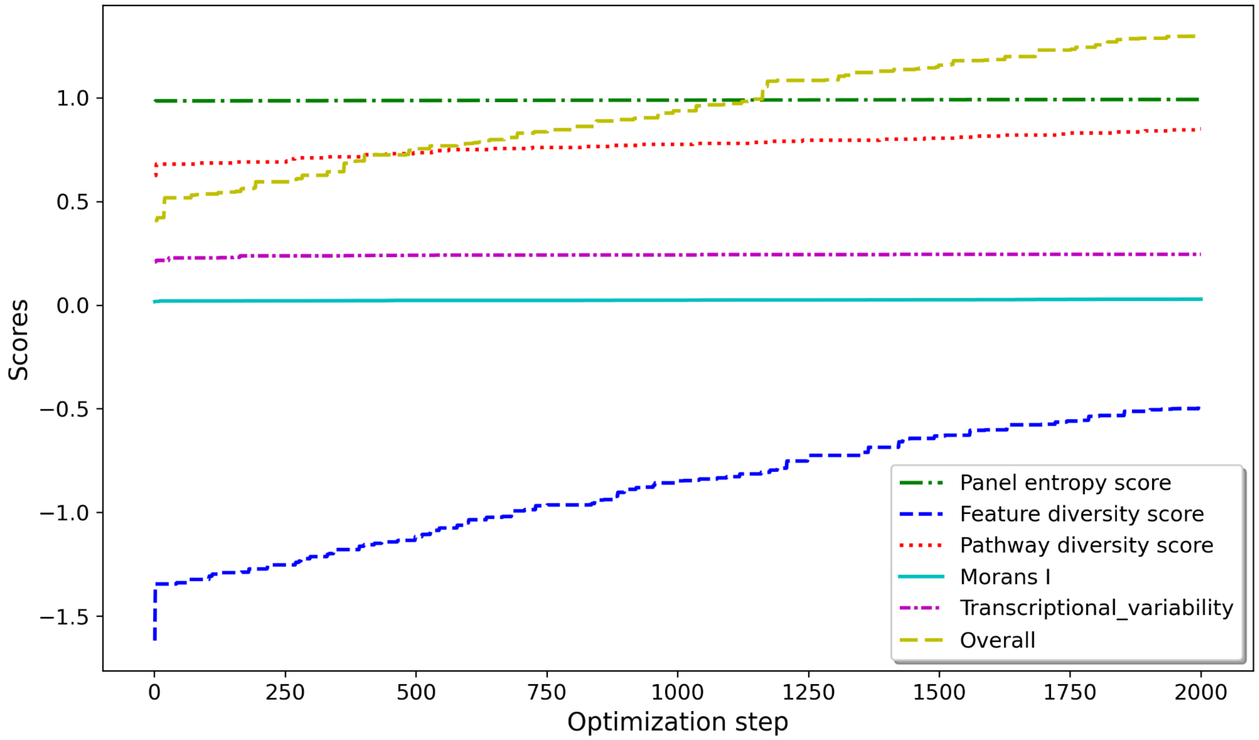

Supplementary Figure 2. Metric changes over the course of genetic algorithm iterations. The plot shows the progress of five objective functions— Panel entropy score, Feature diversity score, Pathway diversity score, Moran's I and transcriptional-variability-based—tracked against their overall metric. The y-axis represents the score values, while the x-axis represents the optimization steps. All metrics show steady increases in score throughout the optimization process, indicating improved performance with continued iterations. Some flat lines are only due to the drawing reasons (the range of y-axis) and would show increase when zoomed in (exemplified as Supplementary 3).
