# Supplementary material for: Multi-view gene panel characterization for spatially resolved omics
Source: Brief Bioinform. 2025 Oct 4;26(5):bbaf478. doi: 10.1093/bib/bbaf478 (PMC12495993; doi:10.1093/bib/bbaf478)
Supplement: Supplementary_figure_3_bbaf478 [file supplementary_figure_3_bbaf478.pdf]

Supplementary Figure 3

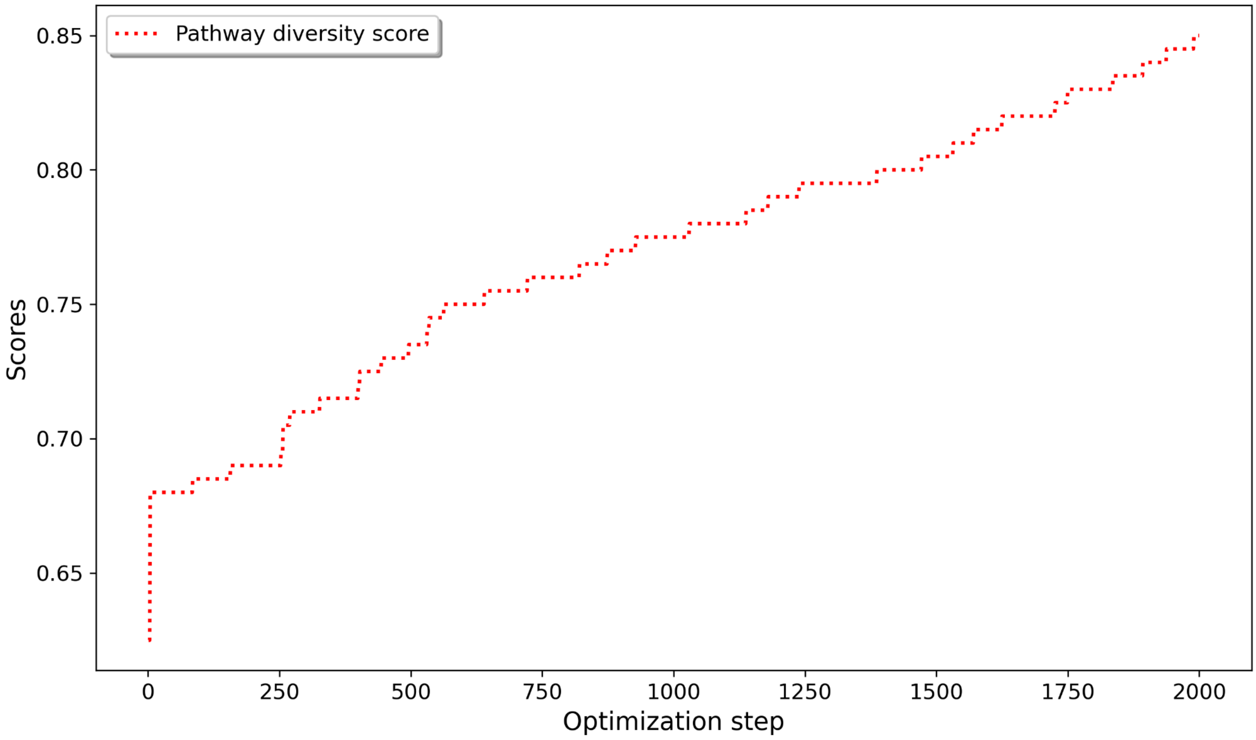

Supplementary Figure 3. Pathway diversity score changes over the course of genetic algorithm iterations with a zoomed-in perspective mentioned in Supplementary figure 2.
