# Supplementary material for: Multi-view gene panel characterization for spatially resolved omics
Source: Brief Bioinform. 2025 Oct 4;26(5):bbaf478. doi: 10.1093/bib/bbaf478 (PMC12495993; doi:10.1093/bib/bbaf478)
Supplement: Supplementary_figure_4_bbaf478 [file supplementary_figure_4_bbaf478.pdf]

Supplementary Figure 4

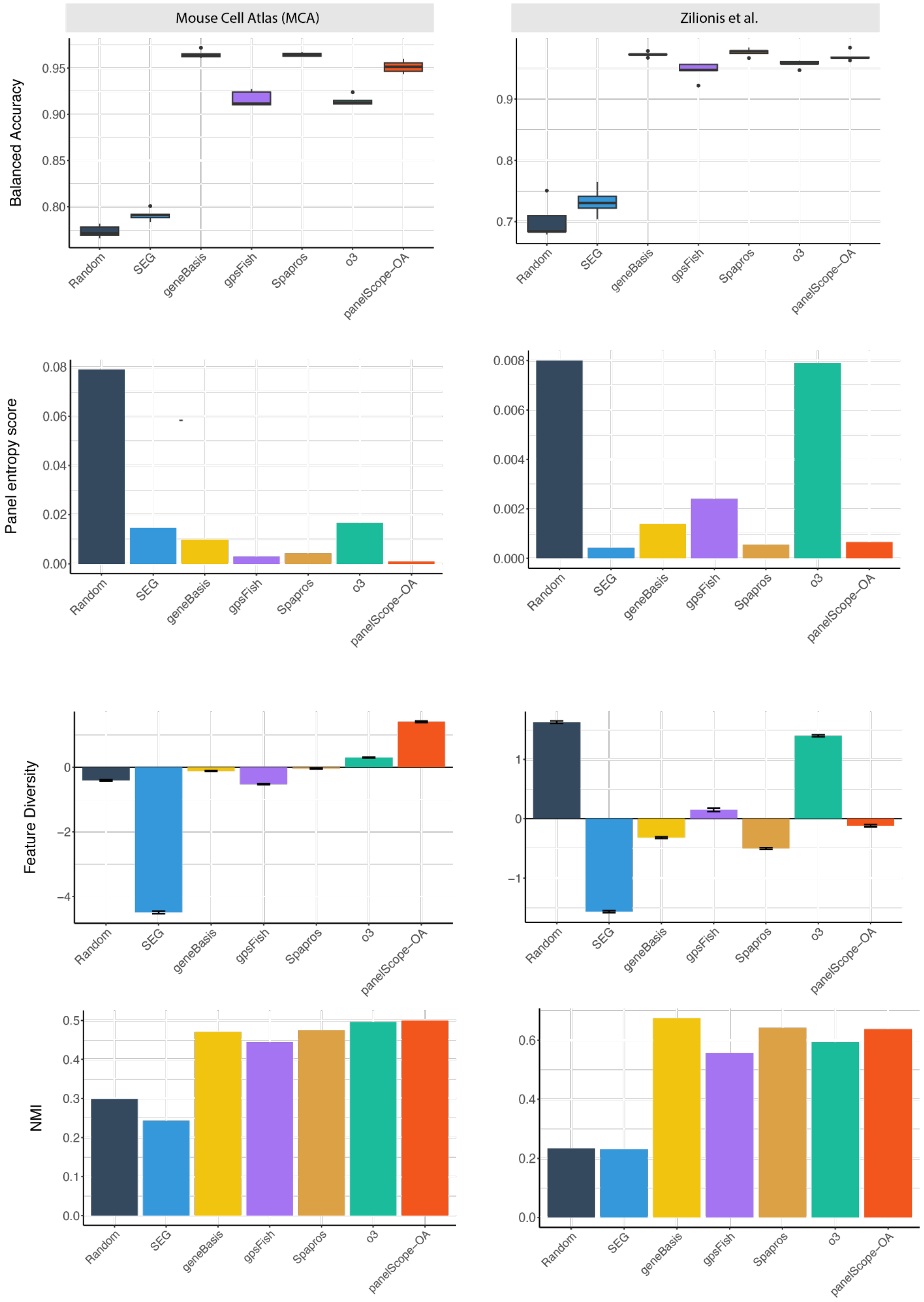

Supplementary Figure 4. Benchmarking of gene-panel design methods on three datasets: the Mouse Cell Atlas (10x Genomics) [1] and lung single-cell data from Zilionis *et al.* [2]. Owing to time constraints, we did not construct a manually curated "Expert" panel. All analyses use the most recent OpenAI model, o3, which supersedes the previously employed o1. Please note that *panelScope-OA* is explicitly trained to maximise the very metrics reported by panelScope. Consequently, any head-to-head comparison with other design tools—whose optimisation

objectives differ—will necessarily favour our method. The results should therefore be interpreted with this caveat in mind.

Ref:
[1] Schaum et al., Single-cell transcriptomics of 20 mouse organs creates a Tabula Muris. Nature, 2018.
[2] Zilionis et al., Single-Cell Transcriptomics of Human and Mouse Lung Cancers Reveals Conserved Myeloid Populations across Individuals and Species. Immunity, 2019.
