# Supplementary material for: Multi-view gene panel characterization for spatially resolved omics
Source: Brief Bioinform. 2025 Oct 4;26(5):bbaf478. doi: 10.1093/bib/bbaf478 (PMC12495993; doi:10.1093/bib/bbaf478)
Supplement: Supplementary_figure_5_bbaf478 [file supplementary_figure_5_bbaf478.pdf]

Supplementary Figure 5

MCA

Panel	# genes	% genes	# pathways	max q-value	redundancy score
Random	0	0	0	-	-
o3	183	0.915	2235	0.02	0.74
Spapros	154	0.77	406	0.038	0.49
SEG	150	0.75	138	0.043	0.652
geneBasis	156	0.78	473	0.036	0.514
gpsFish	144	0.72	233	0.04	0.524
panelScope-OA	102	0.51	258	0.039	0.453

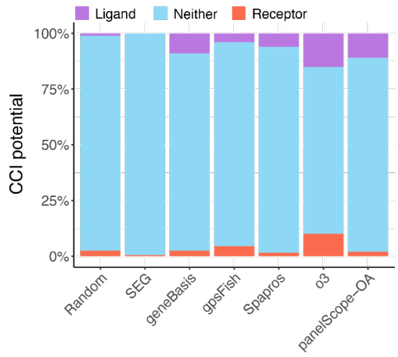

Zilionis et al.

Panel	# genes	% genes	# pathways	max q-value	redundancy score
Random	0	0	0	-	-
o3	165	0.825	982	0.03	0.664
Spapros	132	0.66	638	0.035	0.644
SEG	150	0.75	138	0.043	0.652
geneBasis	141	0.705	644	0.035	0.632
gpsFish	79	0.79	252	0.04	0.563
panelScope-OA	112	0.56	637	0.035	0.633

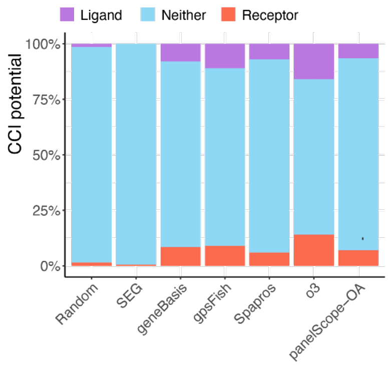

5k Xenium Panel (Human lymph node)

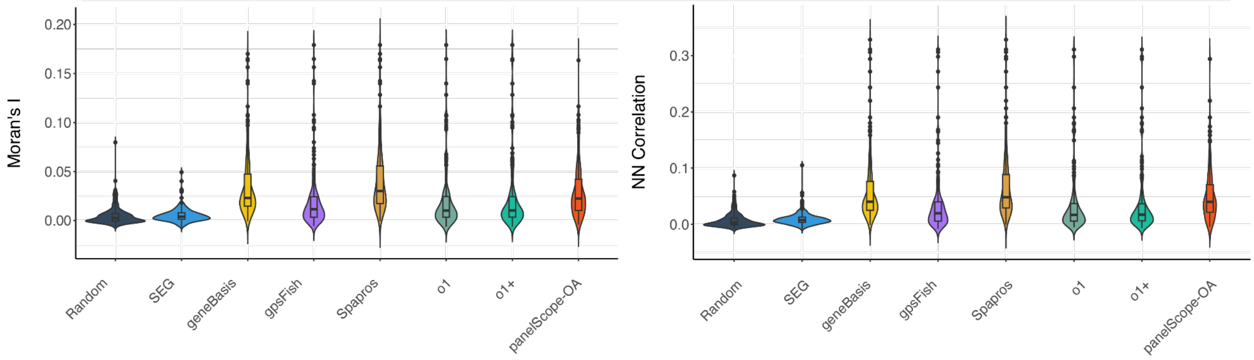

Supplementary Figure 5. Continuation of benchmarking results from Supplementary figure 4. Including results from the 5k Xenium spatial panel from 10x Genomics [1]. For the 5 k Xenium panel, the benchmark is identical to that presented in the main manuscript except that it now includes the panel generated by *panelScope-OA*.

Ref:

[1] https://www.10xgenomics.com/resources/datasets
