# Supplementary material for: Multi-view gene panel characterization for spatially resolved omics
Source: Brief Bioinform. 2025 Oct 4;26(5):bbaf478. doi: 10.1093/bib/bbaf478 (PMC12495993; doi:10.1093/bib/bbaf478)
Supplement: Supplementary_material_1_bbaf478 [file supplementary_material_1_bbaf478.pdf]

Supplementary material 1

Feature specificity

This category aims to assess a panel's ability to distinguish groups of interest (e.g., cell types or cell states). We evaluate the performance of a random forest model trained on a dataset subsetted by a given panel's genes. If the genes in a given panel are informative, we expect the model to perform well in identifying these groups. We quantify this using balanced accuracy and accuracy stratified by the groups of interest as metrics.

Additionally, we assess the cell type specificity of a gene panel of size G, which provides a measure of how uniquely a gene is expressed in a cell type relative to all other cell types. Suppose we aim to capture the set of all cell types C in our panel design, a natural uniqueness score is the measure the abundance of cells within a given cell type $c \in C$ that express the gene relative to all other cell types $c' \in C$.

Gene specificity score (*gsc*) - This score aims to quantify the specificity of an individual gene for a given cell type. For a typical gene g, let us denote x_{cj} as the expression for the *jth* cell in cell type $c \in C$, n_c and $n_{c'}$ represents the total number of cells in cell type c and all other cell types c' respectively. We define the gene specificity score for gene g in cell type *c* as:

$$gene\ specificity\ score\ (gsc) = log(\frac{1}{n_c}\sum_{j=1}^{n_c} I(x_{cj} > 0) + 0.01)\ - log(\frac{1}{n_{c'}}\sum_{k=1}^{n_{c'}} I(x_{c'j} > 0) + 0.01),$$

where $I(x > 0)$ is an indicator function and equals 1 if $x > 0$, and 0 otherwise. Note that we typically have an arbitrary small value of 0.01 inside the logarithm to avoid taking the log of zero. A large specificity score indicates that gene g is a unique marker for cell type c, with a higher proportion of cells expressing the gene of interest in cell type c relative to all other cell types c'. In contrast, a negative specificity score suggests that the gene is expressed in a higher proportion of the other cell types c' relative to cell type c. A score close to zero, suggests that the gene is expressed in equal proportion to cell types c and c'.

Panel entropy score (*pes*) - This score aims to quantify the overall specificity of gene panel specificity for the cell types in a dataset by measuring spatial autocorrelation of the gsc scores after hierarchical clustering, weighted by the proportion of zeros. Here, a mosaic pattern arising from a low proportion of zeros and higher gsc scores are desirable. Such patterns indicate that the genes in a panel are predominantly cell type specific. To summarize this information, we calculate a weighted Moran's I, which quantifies spatial autocorrelation across $gsc, c \in C \text{ and } g \in G$ for all genes and cell types. Where *N* is the number of log ratios indexed by *i* and *j*; $\overline{gsc}$ is the mean of the gsc; w_{ij} are the elements of the spatial weights with zeroes on the diagonal (i.e., $w_{ij} = 0$), where we use the standardized rows of the gsc matrix as the weights; and *W* is the sum of all w_{ij}.

$$Moran's\ I = \frac{N}{W} \frac{\sum_{i=1}^{N} \sum_{j=1}^{N} w_{ij}(gsc_i - \overline{gsc})(gsc_j - \overline{gsc})}{\sum_{i=1}^{N}(gsc_i - \overline{gsc})^2}$$

Finally, we weight Moran's I by the proportion of zeros p' to compute the panel entropy score for a given gene panel:

$$panel\ entropy\ score(PES) \ = \ p' \cdot Moran's\ I$$

Variation recovery score - To measure a gene panel's ability to recover transcriptional variation, we compute Normalized Mutual Information (NMI) between cluster assignments from the full dataset and a subset defined by the panel using Seurat (v5.1.0). A high NMI indicates that the panel effectively preserves transcriptional information, distinguishing cell types and states despite reduced gene dimensionality. Specifically, cells are clustered twice: once with all genes (Clustering A) and once using only the selected panel genes (Clustering B). NMI quantifies the similarity between these two clusterings:

$$NMI(A, B) \ = \ \frac{MI(A,B)}{\frac{1}{2}(H(A)+H(B))},$$

where A and B is the clustering results for the full dataset and subsampled dataset, respectively; MI is the mutual information between clustering A and clustering B; $H(A)$ and $H(B)$ are the entropy of clusterings A and B, respectively. Mutual information quantifies the amount of information shared between the two clusterings A and B, and is defined as:

$$MI(A,B) = \sum_{i=1}^{|A|}\sum_{j=1}^{|B|}\frac{|A_i \cap B_j|}{N}log(\frac{N|A_i \cap B_j|}{|A_i||B_j|}),$$

where $|A|$ and $|B|$ is the total number of clusters in clustering A and B, respectively; $|A_i|$ is the number of cells in cluster i of clustering A; $|B_j|$ is the number of cells in cluster j of clustering B; $|A_i \cap B_j|$ is the intersection of cells between cluster i in A and cluster j in B; N represents the total number of cells across both clusterings.

The entropy of cluster A is given by:

$$H(A) = -\sum_{i=1}^{|A|}\frac{|A_i|}{N}log(\frac{|A_i|}{N}),$$

and similarly for $H(B)$.

Feature diversity

Feature diversity score (*fds*) - To maximize the informativeness of a gene panel, we assess redundancy by computing pairwise Spearman's correlations among all genes. For a panel with G genes, we calculate $G(G-1)/2$ gene pairs and use these to compute the feature diversity score (fds), which reflects the ratio of negative/zero- to positively correlated pairs. A higher fds indicates lower redundancy and greater diversity. A constant of 1 is added to correlation values to avoid taking the log of zero. The feature diversity score is defined below:

$$fds = log(\sum_{i=1}^{G'} I(p_i \leq 0) + 1) - log(\sum_{i=1}^{G'} I(p_i > 0) + 1),$$

a higher fds score indicates greater feature diversity within a panel, whereas a lower fds score suggests reduced diversity and thus higher redundancy. We also provide Woolf-adjusted standard errors to quantify sampling variability. These SEs are calculated under the assumption that features assigned to the non-positive-correlation group and those in the positive-correlation group are independent.

Biological inference

Pathway diversity score (*pds*)- We summarize pathway information in gene panels using over-representation analysis with the enrichGO() function from the clusterProfiler package (v4.10.1). Significant pathways are identified with an adjusted p-value < 0.05. For each panel, we report the count and proportion of genes enriched in significant pathways, the total number of these pathways, the maximum q-value (FDR), and a pathway diversity score (pds) reflecting the variety of pathways represented.

The pds is calculated as the one minus the proportional reduction in the number of pathways after removing pathways with a Jaccard similarity index greater than 0.7. A higher diversity score indicates that the gene panel contains a large proportion of diverse pathways, where P is the total number of significant pathways for the gene panel; P' is the number of significant pathways remaining after removing pathways with a Jaccard similarity index <0.7; p_i is the contribution of pathway i to the total pathway set, and p_j is the contribution of pathway j to the total set of pathways remaining after removing pathways with a Jaccard similarity index greater than 0.7:

$$pds = 1 - \frac{\sum_{i=1}^{P} p_i - \sum_{j=1}^{P'} p'_j}{\sum_{i=1}^{P} p_i}$$

Cell-Cell interaction information - To evaluate the potential for cell–cell interactions within a gene panel, we first classify each gene as either a ligand or a receptor according to annotations from the CellChat database [17], which provides a comprehensive catalog of known ligand–receptor pairs across diverse cell types and

biological systems. We then sum the number of genes in each category—ligands and receptors—to determine how enriched the panel is for each.

Spatial information

Moran's I - We capture the spatial autocorrelation of all genes within a panel by calculating Moran's I for each gene g. Similar to above, let x_g represent the expression of gene g; *N* is the number of expression values indexed by *i* and *j*; $\overline{x_g}$ is the mean expression of gene g across cells ; w_{ij} are the elements of the spatial weights with zeroes on the diagonal (i.e., $w_{ij} = 0$), where we use the standardized rows of the *log ratio* matrix as the weights; and *W* is the sum of all w_{ij}. Moran's I for a gene g is defined below:

$$Moran's\ I(g) = \frac{N}{W}\frac{\sum_{i=1}^{N}\sum_{j=1}^{N} w_{ij}({x^g}_i - \overline{x^g})({x_j}^g - \overline{x^g})}{\sum_{i=1}^{N}({x^g}_i - \overline{x^g})^2}$$

Nearest neighbour correlation - We calculate the correlation of the gene expression values for a panel's genes between a cell and its nearest neighbours, using the package *scFeatures* (version 1.3.4). Briefly, scFeatures calculates the nearest neighbour correlation for each gene within a gene panel. This is calculated by taking the correlation between each cell and its nearest neighbours for a particular gene.

Forward-compatibility

A crucial aspect of gene panel design is ensuring the selected genes remain relevant across various conditions, including treatments, experimental groups, or perturbations—termed "future versatility" or "forward compatibility". To evaluate this, we employed the GEARS perturbation simulator [18], trained on multiple public single-cell perturbation datasets [19–22]. Datasets were processed according to GEARS guidelines, involving normalization and quality filtering. The trained GEARS model simulated perturbations of each gene in the training set, focusing on overlapping genes between the training data and our panel. This approach allowed us to assess the sensitivity of panel genes effectively. We summarized each gene's

perturbation impact by calculating a perturbation score, reflecting the number and magnitude of influenced genes.

Let d represent a G'xG matrix of predicted absolute expression values for G' target genes which are influenced by G perturbed genes, calculated from training and testing on D datasets. The q-value represents the 25th percentile of the gene expression values x in D. The perturbation potential for a gene g is defined as:

$$perturbation\ potential = \frac{\sum_{i=1}^{G} w_i \cdot \sum_{j=1}^{G\prime} (x_{ij} | x_{ij} > q)}{D},$$

where w_i is a weight capturing the proportion of target genes influenced by gene i and have non-zero expression. This metric captures the magnitude of gene expression changes induced by target genes, normalized by the number of genes they influence. A higher perturbation potential indicates that a gene is more likely to influence the expression of other genes when perturbed. To calculate the perturbation potential of a panel, we simply take the mean of the perturbation potential scores across the genes.

Gene importance score and overall score

Gene importance score - To provide a comprehensive characterization of the gene panels, A we compute an importance score for each gene within a panel, which reflects the gene's contribution to a panel across the defined categories. Before computing the scores, we standardize each individual component from the broad categories to ensure comparability. These components are then equally weighted by default; however, users can choose to assign different weights to the components based on their specific research questions or priorities.

For a typical gene g, we define the gene importance score (gis) as

$$Gene\ importance\ score_g = w_1 \cdot rf_g + w_2 \cdot FDS_g\ + w_3 \cdot k_g$$

Where rf_g is the importance score for gene g output from the random forest model used for cell type classification; fds_g is the feature diversity score; and k_g is an indicator variable, such that $k = 1$ if gene g is enriched in a pathway, 0 otherwise; and w_1, w_2, w_3 are weights which can be user-specified to allow for customization based on the importance of each metric. By default, all weights are set to 0.33. Thus, gene importance scores within a panel are comparable but should not be compared across panels.

Weighted overall score - For a given panel $P = \{g_1, g_{2,} \ldots, g_G\}$, the weighted overall panel score is define as

$$Weighted\ overall\ score(G) = w_1 \cdot\ Balanced\ accuracy_G + w_2 \cdot (1 - pes_G) +\ w_3 \cdot fds_G + w_4 \cdot \overline{k_G},$$

where *balanced accuracy* represents the balanced cell type classification accuracy for gene panel G; pas is the panel entropy score; fds is the feature diversity score; $\overline{k_g}$ is the mean of the binary variable k, which indicates whether each gene in a panel is enriched in a significant pathway (k=1) or not (k=0). Since k is binary, $\bar{k}$ corresponds to the proportion of genes in the panel enriched in significant pathways; and w_1, w_2, w_3, w_4 are weights which can be user-specified to allow for customization based on the importance of each metric. By default, all weights are set to 0.25.

panelScope-OA pseudocode for panel search algorithm

Algorithm 1. Evolutionary Panel Search

Definitions:
– individual: each panel is one individual, containing 200 genes;
– population: 50 gene panels (individuals) construct the population, all our evolutionary algorithm is then operated on this population.

Inputs:
- search_space — map of positions → candidate genes
- pop_size — number of individuals
- max_concurrency — max trials in flight
- objmode — optimize_mode

```
1. Initialize:
  population ← pop_size random configs sampled from search_space
  running ← Ø

2. Loop until stopping condition:
  a. Dispatch new trials (up to max_concurrency):
    for each slot free in running:
      if ∃ config ∈ population with no result:
        indiv ← take one unevaluated config
      else:
        parent ← best(population)
        indiv ← mutate(parent, search_space)
      mark indiv as running, submit for evaluation

  b. Collect completed trials:
    for each indiv in running that finished:
      reward ← compute_reward(indiv)  // sum or specific metric
      if optimize_mode == "minimize": reward ← –reward
      store result in indiv
      running ← running \ {indiv}

  c. Update population:
    add evaluated indivs back into population
    if population > pop_size:
      remove worst individuals to keep size

3. Return best configuration found
```
