# Supplementary material for: Multi-view gene panel characterization for spatially resolved omics
Source: Brief Bioinform. 2025 Oct 4;26(5):bbaf478. doi: 10.1093/bib/bbaf478 (PMC12495993; doi:10.1093/bib/bbaf478)
Supplement: Supplementary_material_2_bbaf478 [file supplementary_material_2_bbaf478.pdf]

Supplementary material 2

Prompt 1

I am designing a gene panel consisting of 200 genes in the context of Acute Myeloid Leukemia (AML). The goal is to optimize the panel based on the following criteria:

- Cell type specific markers to capture major and minor cell types in AML.
- Transcriptional variation, try to capture as much of the transcriptional variation possible.
- Feature diversity, ensuring minimal redundancy among positively correlated features.
- Inclusion of diverse pathways to capture broad biological relevance.
- Representation of ligands and receptors, crucial for cell-cell communication.
- Inclusion of spatially variable genes, capturing spatial heterogeneity in AML.

Please design a gene panel of 200 genes based on these criteria. Provide the gene list in one continuous line, with each gene enclosed in quotation marks and separated by commas.

Response 1

“CD34”,”KIT”,”FLT3”,”GATA2”,”RUNX1”,”MECOM”,”MEIS1”,”HOXA9”,”BMI1”,”EZH2”,
“MPO”,”CEBPA”,”CEBPE”,”SPI1”,”CSF1R”,”CSF3R”,”ITGAM”,”LYZ”,”S100A8”,”S100A9”,
“CD14”,”FCGR3A”,”ITGAX”,”CD68”,”MRC1”,
“GYPA”,”HBB”,”HBA1”,”ALAS2”,”KLF1”,”EPOR”,”SLC4A1”,”FECH”,
“ITGA2B”,”GP1BA”,”PF4”,”NFE2”,”TUBB1”,
“MS4A1”,”CD19”,”CD79A”,”CD79B”,”PAX5”,”EBF1”,”IGHM”,”IGHD”,”CD22”,”VPREB1”,
“CD3D”,”CD3E”,”CD3G”,”CD4”,”CD8A”,”CD8B”,”CD2”,”CD5”,”CD7”,”CD28”,”CTLA4”,”PDCD1”,”ICOS”,”TRAC”,”TRBC1”,
“NCR1”,”KLRD1”,”KLRC1”,”KLRC2”,”KIR2DL3”,”KIR3DL1”,”GNLY”,”NKG7”,”PRF1”,”GZMB”,
“CD1C”,”CLEC9A”,”XCR1”,”IRF8”,”BATF3”,”FCER1A”,”CLEC10A”,”SIRPA”,
“SDC1”,”IRF4”,”PRDM1”,”XBP1”,
“LILRA4”,”IL3RA”,”TCF4”,”SPIB”,”IRF7”,
“GATA1”,”IL5RA”,”CPA3”,”PRG2”,”KITLG”,
“NPM1”,”DNMT3A”,”TET2”,”IDH1”,”IDH2”,”KRAS”,”NRAS”,”TP53”,”CBFB”,”WT1”,”KMT2A”,”MLLT3”,”PML”,”RARA”,”SF3B1”,
“CD40”,”CD40LG”,”CXCL12”,”CXCR4”,”PDCD1LG2”,”CD274”,”CD80”,”CD86”,”ICAM1”,”VCAM1”,”SELE”,”SELP”,”SIRPB1”,”MICA”,”MICB”,”NOTCH1”,”JAG1”,”DLL1”,”TGFB1”,”TGFBR1”,”TGFBR2”,
“WNT3A”,”WNT5A”,”FZD1”,”CTNNB1”,”AXIN1”,
“JAK2”,”STAT3”,”STAT5B”,”SOCS1”,”SOCS3”,
“CD44”,”VIM”,”FN1”,”MMP9”,”ANGPT1”,”ANGPT2”,”COL1A1”,”COL3A1”,”LAMC2”,”ITGB1”,
“MYB”,”GFI1”,”BCL11A”,”FOXO3”,”CEBPD”,”IRF1”,”ETS1”,”IKZF1”,”NFIA”,”RUNX2”,
“BCL2”,”MCL1”,”BAX”,”BAK1”,”CASP3”,”CASP8”,”CDKN1A”,”CDKN2A”,”MDM2”,”RB1”,
“GAPDH”,”LDHA”,”PFKP”,”HK1”,”G6PD”,”IDH3A”,”SDHA”,”NDUFA9”,”COX4I1”,”ATP5F1B”,

“CXCL8”,”CCL2”,”CCL3”,”CCL5”,”IL1B”,”IL6”,”TNF”,”CD70”,”TNFRSF1A”,”IL10”, “ASXL1”,”BCOR”,”PHF6”,”ARID1A”,”SMARCA4”,”CREBBP”,”EP300”,”CBX5”,”SUZ12”

Prompt 2

I am designing a gene panel consisting of 200 genes in the context of Acute Myeloid Leukemia (AML). The goal is to optimize the panel based on the following criteria:

- Cell type specific markers, where the cell types of interest are: Aberrant erythroid, CD4+ cytotoxic T cells, CD4+ memory T cells, CD4+ naive T cells, CD56brightCD16- NK cells, CD56dimCD16+ NK cells, CD69+PD-1+ memory CD4+ T cells, CD8+ central memory T cells, CD8+ effector memory T cells, CD8+ naive T cells, CD8+CD103+ tissue resident memory T cells, Class switched memory B cells, Classical Monocytes, Conventional dendritic cell 1, Conventional dendritic cell 2, Early erythroid progenitor, Early promyelocytes, Eosinophil-basophil-mast cell progenitors, Erythro-myeloid progenitors, GammaDelta T cells, HSCs & MPPs, Immature B cells, Late erythroid progenitor, Late promyelocytes, Lymphomyeloid prog, Mature naive B cells, Megakaryocyte progenitors, Myelocytes, Non-classical monocytes, Nonswitched memory B cells, Plasma cells, Plasmacytoid dendritic cell progenitors, Plasmacytoid dendritic cells, Pre-B cells, Pre-pro-B cells, Pro-B cells, Small pre-B cell
- Transcriptional variation, try to capture as much of the transcriptional variation possible.
- Feature diversity, ensuring minimal redundancy among positively correlated features.
- Inclusion of diverse pathways to capture broad biological relevance.
- Representation of ligands and receptors, crucial for cell-cell communication.
- Inclusion of spatially variable genes, capturing spatial heterogeneity in AML.

Please design a gene panel of 200 genes based on these criteria. Provide the gene list in one continuous line, with each gene enclosed in quotation marks and separated by commas.

Response 2

“GYPA”,”HBB”,”HBA1”,”KLF1”,”EPOR”,”CD4”,”GZMB”,”PRF1”,”FASLG”,”EOMES”,”CD4”,”CD44”,”IL7R”,”CD28”,”CXCR5”,”CD4”,”TCF7”,”CCR7”,”LEF1”,”SELL”,”NCAM1”,”NKG7”,”IL2RB”,”ZEB2”,”KIT”,”NCAM1”,”FCGR3A”,”PRF1”,”GNLY”,”KIR2DL3”,”CD4”,”CD69”,”PDCD1”,”IL2RA”,”CXCR3”,”CD8A”,”TCF7”,”IL7R”,”SELL”,”CD27”,”CD8A”,”GZMA”,”GZMB”,”PRF1”,”GNLY”,”CD8A”,”CCR7”,”LEF1”,”TCF7”,”PTPRC”,”CD8A”,”ITGAE”,”CD69”,”ITGA1”,”CXCR6”,”CD19”,”MS4A1”,”AICDA”,”CD27”,”IGHG1”,”CD14”,”LYZ”,”S100A8”,”S100A9”,”FCGR1A”,”CLEC9A”,”XCR1”,”BATF3”,”IRF8”,”CADM1”,”CD1C”,”FCER1A”,”CLEC10A”,”SIRPA”,”IRF4”,”GATA1”,”TAL1”,”KLF1”,”AHSP”,”ALAS2”,”MPO”,”CD33”,”CEBPA”,”GFI1”,”LYZ”,”GATA2”,”PRG2”,”CPA3”,”IL5RA”,”ENPP3”,”CD34”,”GATA2”,”GATA1”,”CSF1R”,”SPI1”,”TRDV1”,”TRDV2”,”TRGC1”,”TRGC2”,”CD3D”,”CD34”,”KIT”,”FLT3”,”MEIS1”,”HOXA9”,”CD19”,”IGM”,”IGHD”,”VPREB1”,”PAX5”,”GYPA”,”FECH”,”SLC4A1”,”TMOD1”,”GATA1”,”MPO”,”CEBPE”,”PRTN3”,”ELANE”,”CD33”,”MEF2C”,”DNTT”,”RAG1”,”RAG2”,”IL7R”,”CD19”,”MS4A1”,”IGHD”,”CR2”,”BANK1”,”ITGA2B”,”GP1BA”,”PF4”,”NFE2”,”TUBB1”,”MPO”,”ELANE”,”CEBPE”,”PRTN3”,”MMP8”,”F

CGR3A”,”NR4A1”,”CX3CR1”,”CD14”,”ITGAL”,”CD19”,”MS4A1”,”CD27”,”FCER2”,”IGHM”,”SLAMF7”,”CD38”,”XBP1”,”PRDM1”,”IRF4”,”TCF4”,”IL3RA”,”LILRA4”,”SPIB”,”IRF8”,”IL3RA”,”LILRA4”,”TCF4”,”IRF7”,”GZMB”,”VPREB1”,”IGLC2”,”IGLL1”,”CD79A”,”EBF1”,”KIT”,”FLT3”,”EBF1”,”TCF3”,”IGLL1”,”PAX5”,”EBF1”,”CD79A”,”CD79B”,”VPREB3”,”VPREB3”,”CD24”,”PAX5”,”RAG1”,”RAG2”,”NPM1”,”IDH1”,”IDH2”,”WT1”,”TP53”,”DNMT3A”,”TET2”,”CBFB”,”KMT2A”,”RARA”,”CXCL12”,”CXCR4”,”CD274”,”PDCD1LG2”,”HLA-DRB1”
