# Supplementary material for: Multi-view gene panel characterization for spatially resolved omics
Source: Brief Bioinform. 2025 Oct 4;26(5):bbaf478. doi: 10.1093/bib/bbaf478 (PMC12495993; doi:10.1093/bib/bbaf478)
Supplement: Supplementary_material_3_bbaf478 [file supplementary_material_3_bbaf478.pdf]

Supplementary material 3

Prompt 1

I am designing a gene panel consisting of 200 genes in the context of lymph node cancer in humans. The goal is to optimize the panel based on the following criteria:

- Cell type specific markers to capture major and minor cell types in lymph node cancer.
- Transcriptional variation, try to capture as much of the transcriptional variation possible.
- Feature diversity, ensuring minimal redundancy among positively correlated features.
- Inclusion of diverse pathways to capture broad biological relevance.
- Representation of ligands and receptors, crucial for cell-cell communication.
- Inclusion of spatially variable genes, capturing spatial heterogeneity in the lymph node.

Please design a gene panel of 200 genes based on these criteria. Provide the gene list in one continuous line, with each gene enclosed in quotation marks and separated by commas.

Response 1

"ACTB","GAPDH","RPL13A","RPS29","RPLP0","TUBB","UBC","YWHAZ","B2M","HPRT1", "MS4A1","CD19","CD79A","CD79B","PAX5","BCL2","BCL6","MYC","PIM1","EZH2", "CREBBP","SOCS1","TNFRSF14","CD22","IRF8","CD3D","CD3E","CD3G","CD4","CD8A", "CD8B","FOXP3","CTLA4","PDCD1","ICOS","TRAC","TRBC1","TRBC2","NCAM1","KLRD1", "NKG7","GNLY","FCGR3A","KIR2DL1","KIR3DL1","GZMB","CD14","CD68","CD163","ITGAX", "CLEC9A","LYZ","FCER1A","CD1C","MARCO","MERTK","TNFRSF8","FUT4","STAT6","JAK2", "REL","NFKB1","PDPN","FAP","COL1A1","COL1A2","DCN","BGN","THY1","VIM", "MMP2","MMP9","PECAM1","VWF","ENG","CD34","ICAM1","IL2","IL4","IL6", "IL7","IL10","IL12A","IL15","IL17A","IL1B","IFNG","TNF","TGFB1","CCL2", "CXCL9","CXCL10","CXCL11","CXCL13","CCL19","CCL21","CXCL12","CXCL14","CCR7","CXCR5", "CD28","SLAMF1","PDCD1LG2","CD274","CD80","CD86","CD40","CD40LG","CD70","CD27", "TNFRSF9","TNFSF9","MKI67","PCNA","CDK1","CDK2","CCND1","CCNE1","CDKN1A","E2F1", "AKT1","AKT2","PTEN","PIK3CA","MAPK1","MAPK3","MAPK8","MAPK14","NFATC1","JAK1", "TYK2","STAT1","STAT3","STAT5A","NFKBIA","RELA","BRAF","KRAS","ERK","SRC", "LDHA","HK2","PKM","GPI","PGK1","PFKP","SLC2A1","SLC16A1","MTOR","AMPK", "CD44","CDH1","CDH2","SNAI1","TWIST1","ZEB1","ZEB2","FN1","SPARC","VCAM1", "DNMT1","TET2","KDM6B","KMT2A","HDAC1","HDAC2","SMARCA4","ARID1A","BRD4","EP300", "TP53","RB1","CDKN2A","ATM","BRCA1","BRCA2","PTPN11","ETS1","FOS","JUN", "CASP3","CASP8","CASP9","BID","BAX","BAK1","MCL1","XIAP","BIRC5","FAS", "TNFAIP3","CARD11","NFKB2","RELB","IRF4","KMT2D","HIST1H1E","MYD88","CD2","IGHM"

Prompt 2

I am designing a gene panel consisting of 200 genes in the context of lymph node cancer in humans. The goal is to optimize the panel based on the following criteria:

• Cell type specific markers, where the cell types of interest are: B cells, plasma cells, monocyte/macrophage, fibroblasts, endothelial cells, and malignant cells.

• Transcriptional variation, try to capture as much of the transcriptional variation possible.
• Feature diversity, ensuring minimal redundancy among positively correlated features.
• Inclusion of diverse pathways to capture broad biological relevance.
• Representation of ligands and receptors, crucial for cell-cell communication.
• Inclusion of spatially variable genes, capturing spatial heterogeneity in AML.

Please design a gene panel of 200 genes based on these criteria. Provide the gene list in one continuous line, with each gene enclosed in quotation marks and separated by commas.

<u>Response 2</u>
"ACTB","GAPDH","RPL13A","RPS29","RPLP0","TUBB","UBC","YWHAZ","B2M","HPRT1",
"MS4A1","CD19","CD79A","CD79B","PAX5","CD22","CR2","BLK",
"PRDM1","IRF4","CD27","CD38","SDC1","MZB1","XBP1","JCHAIN",
"CD14","FCGR3A","CD68","MARCO","CSF1R","ITGAX","SIRPA","CD86",
"PDPN","FAP","PDGFRA","PDGFRB","DCN","COL1A1","COL1A2","CXCL12","CXCL14","BGN","ELN","MMP2","MMP3","THY1","VIM",
"PECAM1","VWF","ENG","TIE1","TEK","CD34","FLT1","KDR","NOS3","ICAM1",
"BCL2","BCL6","MYC","PIM1","EZH2","CREBBP","SOCS1","TNFRSF14","IRF8","FOXP1","NOTCH1","TP53","RB1","CDKN2A","ATM","BRCA1","BRCA2","PTEN","KRAS","NRAS","BRAF","KIT","FLT3","IDH1","IDH2","NPM1","TET2","DNMT3A","RUNX1","CEBPA","MPO",
"IL2","IL4","IL6","IL7","IL10","IL12A","IL12B","IL15","IL17A","IL1B","IFNG","TNF","TGFB1","CXCL9",
"CXCL10","CXCL11","CXCL13","CCL19","CCL21","CCL2",
"CTLA4","PDCD1","CD274","PDCD1LG2","CD80","ICOS","CD40","CD40LG","TNFRSF9","TNFSF9",
"MAPK1","MAPK3","MAPK8","MAPK14","AKT1","AKT2","JAK1","JAK2","STAT3","NFKB1",
"KMT2A","KMT2D","HDAC1","HDAC2","SMARCA4","ARID1A",
"FN1","VCAN","LAMA1","LAMC2","VTN","FDCSP","MADCAM1","LYVE1","ACKR4","CCR5","CXCR5",
"ICAM2","VCAM1","SELE","SELPLG","SELL","TGFBI",
"TNFRSF1A","TNFRSF1B","TNFRSF4","TNFSF4","TNFRSF18","TNFSF18","TNFSF14","CD27","CD70","TNFRSF13B",
"TCF3","LEF1","GATA3","TBX21","RORC","RUNX3","MAF","ETS1","FOS","JUN","CARD11","TCF4","BACH2","E2F2","MDM2","MDM4","ETV6","FOXO1","FOXO3","STAT5A","STAT5B","SMAD2","SMAD3","SMAD4","RAD51","HIF1A","BCL3",
"LDHA","HK2","PKM","GPI","PGK1","PFKP","SLC2A1","SLC16A1","MTOR","PRKAA1"
